# The Impact of the COVID-19 Lockdown on Sugar-Sweetened Beverage Consumption in Children in Saudi Arabia: A Mixed-Methods Study

**DOI:** 10.3390/nu14234972

**Published:** 2022-11-23

**Authors:** Khlood Baghlaf, Dania Bormah, Anwar Hakami, Sara M. Bagher

**Affiliations:** 1 Pediatric Dentistry, Faculty of Dentistry, King Abdulaziz University, Jeddah 21589, Saudi Arabia; 2 Faculty of Dentistry, King Abdulaziz University, Jeddah 21589, Saudi Arabia; 3 Saudi Board Residency Program, King Abdulaziz University, Jeddah 21589, Saudi Arabia

**Keywords:** coronavirus, sugar-sweetened beverages, soft drinks, flavored milk, juices, children, caries, sugar

## Abstract

(1) Background: In 2020, there seems to have been a global shift in lifestyle and eating habits with the emergence of the coronavirus disease 2019 (COVID-19) and the ensuing lockdowns implemented by national governments. This study assessed the impact of the COVID-19 lockdown on SSBs consumption among healthy 6–11 years old children in Saudi Arabia; (2) Methods: This is a mixed-methods study, incorporating a quantitative component, which was a validated Arabic online questionnaire completed by parents, and a qualitative component, involving structured interviews with 10 selected parents using a criterion sampling method; (3) Results: There was a small decrease in consumption reported during lockdown across all SSBs types (soft drinks, n = 58 (13.9%); juices: n = 115 (27.6%); flavored milk: n = 93 (22.3%)). The results showed that with every increase in the dental pain scale there was a positive odd (AOR:0.64; *p* = 0.001) of decreased consumption of SSBs. Several themes related to increase and decrease SSBs consumption emerged; (4) Conclusions: There was a small decrease in SSBs consumption during lockdown reported by parents. Several themes emerged that can be used to strategize against problematic eating behavior, enabling such provisions as family dietary interventions, which target both parents and children.

## 1. Introduction

Childhood is a pivotal phase of life that affects future development and growth, requiring a prioritization of key areas of lifestyle, such as social life, diet, and physical activity toward optimum health outcomes [1,2,3]. During this period, a physically active lifestyle and healthy dietary habits are essential to prevent malnutrition and reduce the risk of disease [4].

Sugar consumption is associated with a number of chronic diseases in children worldwide, including obesity, type 2 diabetes mellitus, and dental caries [5]. The World Health Organization (WHO) recommends a daily free sugar (FS) intake of <10% of the child’s total energy intake [6]. Free sugars, monosaccaride, and disaccaride, can be found naturally in fruits or honey, but are often added to commercial food and drink [7]. Perhaps the main source of FS among children is sugar-sweetened beverages (SSBs) [8]. For present purposes, SSBs can be operationally defined as “any product with added sugar or any other sweetened and produced in the form of ready-to-drink, concentrated, powder, gel, extracts, or any other form that can be transformed into a drink” [9,10]. This includes, but is not limited to, soft drinks, fruit juices and fruit drinks, flavored milks, sports drinks, energy drinks, sweetened waters, coffee, and tea (with sugar added) [11].

Several studies show a direct association between dental caries and sugar consumption [12,13,14]. Dental caries is particularly concerning among children in Saudi Arabia, as the estimated prevalence of dental carries among 6–7 years old children in 2019 was reported to be 89% [15]. In 2017, the General Authority of Zakat and Tax (GAZT) in Saudi Arabia announced the application of a 50% excise tax on all types of SSBs [16]. Recently, a study in 2017 assessed the impact of taxes application on the consumption of soft drinks in Saudi Arabia and a sharp decrease in sale volume was reported [17].

Recently, a cross-sectional study in Riyadh explored the prevalence of dental caries among primary school children to determine possible factors associated with dental caries, and the frequent consumption of SSBs was reported to be significantly associated with dental caries [18]. 

The current worldwide response to the coronavirus disease 2019 (COVID-19) has seen a significant widespread shift in lifestyle, primarily as a result of government lockdowns. The COVID-19 lockdowns resulted in various lifestyle changes that affected social life, working habits, schooling, physical activity, and diet. Many studies report detrimental changes in dietary habits [19,20]. To the best of the authors’ knowledge, no studies have yet been conducted in Saudi Arabia regarding the effects of COVID-19 lockdown on the consumption of SSBs among children. The present study assessed the impact of the national lockdown on sugar-sweetened beverage consumption among healthy 6–11 years olds children in Saudi Arabia and explores factors that may have influenced changes in their sugar-sweetened beverage consumption. We hypothesized that COVID-19 lockdown had no impact on SSBs consumption including the children’s consumption of sugar-sweetened beverages in healthy 6–11 years olds children in Saudi Arabia. 

## 2. Materials and Methods

### 2.1. Study Design

The study used a mixed-methods design (quantitative questionnaire and qualitative interview). It included parents of healthy 6–11 years old children living in Saudi Arabia. The study was conducted in accordance with the Declaration of Helsinki and ethical approval was obtained from the ethics committee at King Abdulaziz University Faculty of Dentistry (KAUFD) (125-11-20). 

The study used a mixed-methods design (quantitative questionnaire and qualitative interview). The first phase of the study involved a validated Arabic online questionnaire (Google Forms) completed by parents between December 2020 and February 2021. The first section of the questionnaire involved validated questions taken from an oral health behavior questionnaire [21]. The questionnaire was made available through social media platforms and was designed to gather data from parents in three large cities of Saudi Arabia (Dammam, Jeddah, Riyadh). Parents were asked to answer for only one of their children (the oldest) that met the inclusion and exclusion criteria.

All parents of healthy children aged 6–11 years who agreed to participate in the study and signed a consent form were included. Parents of medically compromised children or children on daily medication were excluded. In the quantitative phase, parents were categorized based on their children SSBs consumption. Then, we proceeded with criterion sampling for the qualitative phase and only parents who reported a decrease or an increase in their children SSBs consumption were selected for the interviews. Those that agreed to participate in the second phase of the study (qualitative interview) were asked to provide contact information in case they were randomly selected for interview.

### 2.2. Sample Size and Sampling Technique

The study incorporated a purposive sample of 417 participants who signed the electronic consent forms and agreed to answer the questionnaires. Sample size is determined using the G.power software [22] (3.1.9.4). Sample size was estimated assuming a 95% confidence level, with a minimum test power of 0.85, and a significance level of 5% (α = 0.05) for a small effect size (0.2). Based on the change in dietary habits during COVID-19 lockdown in a previous analysis of a cross-sectional study by Sidor and Rzymski in 2020. It was estimated that 378 sample size will be adequate [19]. A purposive criterion sampling was used to select parents for qualitative interviews. To calculate sample size, the analysis of data was carried out concurrently with data collection; once data saturation was reached, interviews were ceased. We predicted that approximately 10 interviews were required to reach data saturation [23]. 

### 2.3. First Phase: Questionnaire

In the first phase of the study, participants were required to complete an online Arabic questionnaire that included sociodemographic (nationality, mother and father level of education, the number of children in the family, family average monthly income, the age of the participating parent, and with whom does the child live) and dental health-related questions (history of dental pain within the last year, and visiting the dentist within the last year) from a validated oral health behaviors questionnaire (OHBQ) [21]. The questionnaire was uploaded to the SurveyMonkey online platform and the link was circulated online in several cities in the Kingdom using a snowballing technique. The questionnaire included questions regarding any changes in the child’s consumption of SSBs during lockdown, and possible factors that may have influenced changes in their eating behavior (Appendix A).

Average monthly family income was divided into three levels based on the central statistics and demographic information obtained from a national government website in Saudi Arabia [24]. The second section included questions regarding children’s daily con-sumption of SSBs prior to and during the COVID-19 lockdown. The history of dental pain within the last year was based on the frequency (no pain, once a year, twice a year, three times a year, more than three times a year). 

Ten parents of 6–11 years old healthy children were asked to read the questions and to offer their opinions for face validity. After addressing all the changes based on the face validity, two expert pediatric dentists consultants at KAUFD assessed the questions for experts’ opinions. Finally, after addressing all the comments based on face validity and experts’ opinions, five pediatric consultants individually assessed each question based on the clarity, simplicity, the ambiguity of the questions, and the relevance to the aim of the study on a four-point scale ranging from one to four, with four being the highest. The validity of the questionnaire was evaluated by calculating the content validity index (CVI). 

Following participant response in this phase, those that reported either a decrease or increase in their child’s consumption of SSBs during lockdown were considered qualified for the second phase (qualitative interview).

### 2.4. Second Phase: Interviews

**Figure 1:**
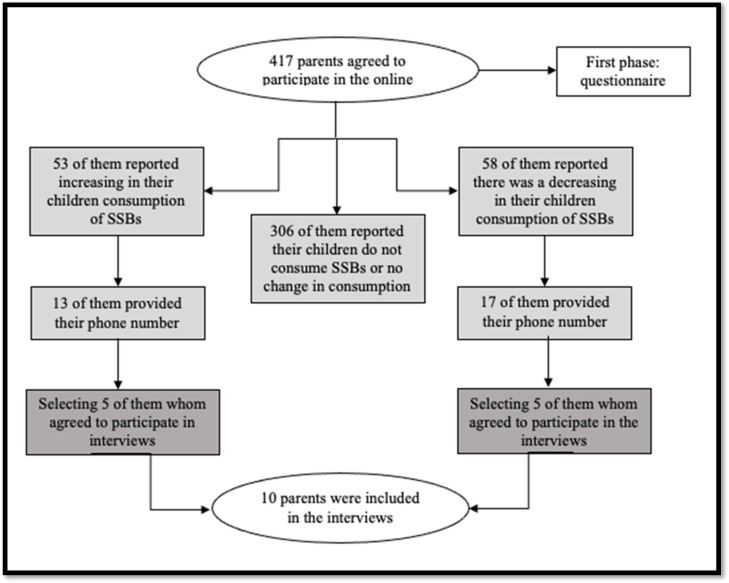
The flow-chart of participants at different research phases (quantitative and qualitative phase).

The second phase involved 10 randomly selected parents that reported changes in their child’s SSBs consumption during lockdown. The parents were divided into two groups: the first group comprised participants that reported an increase in consumption and the second group involved participants that reported a decrease. Five participants were randomly selected from each group. The interviews were conducted with the aid of a smart phone, and followed a structured topic guide (Appendix B). Figure 1 shows the flow-chart of participants at different research phases. The interview incorporated open-ended questions about the child’s SSBs consumption and explored factors that may have influenced changes in their eating behavior, such as parental awareness, financial issues, and lifestyle changes. The interviews were audio-recorded and transcribed verbatim. The interviews were conducted in the Arabic language and translated into English.

### 2.5. Data Analysis

Participants completed an online questionnaire and data were compiled on a Microsoft Excel spreadsheet (version 16.52, Bill Gates and Paul Allen, Albuquerque, New Mexico). All data were anonymized and saved on a password-protected desktop computer. The data were transferred into and analyzed using the Statistical Package for the Social Sciences (SPSS Inc., version 22.0, Chicago, IL, USA). The descriptive analysis included mean (SD), frequency, outcome (change in SSBs consumption), and independent variables (sociodemographic and dental health-related variables). Bivariate analysis was conducted between the sociodemographic factors, dental health factors and the change in SSBs consumption using Chi-square test and Fishers exact test. Correlation was measured between history of dental pain during last year and SSBs consumption using Pearson correlation test. Two binary logistic regression models were conducted for increase and decrease in SSBs consumption and the reference category was no change in SSBs consumption. Multivariate regression analysis was conducted between the categorical outcome (SSBs consumption) and the predictor, which is the child’s history of pain during the last and it was adjusted for all sociodemographic variables and other factors (visiting the dentist within last year). Significance was set at the 5% level.

For qualitative data, iterative data gathering, and analysis were used to reach thematic saturation. The transcripts were made using the technique of intelligent transcription. The researcher listened to each interview and reviewed the transcripts for errors before uploading the transcripts into the software for analysis. Data were analyzed through a thematic analysis with the NVivo qualitative data analysis software (Version 11, 2016, QSR International Pty Ltd., Burlington, Australia). Multiple coding was performed independently by two researchers (KB, DB), who also performed the thematic analysis. Any disagreement of coding was discussed between the two researchers, resulting in a final coding of themes upon reaching consensus. 

## 3. Results

### 3.1. Results of the First Phase (Questionnaire)

The chosen five experts rated the item content of the data collection tool; further, agreement was recorded at 92.5% for the questionnaire parts in the English version. For the Arabic version, the item content of the data collection tool and agreement was tested to record a 95.5% agreement. 

**Figure 2:**
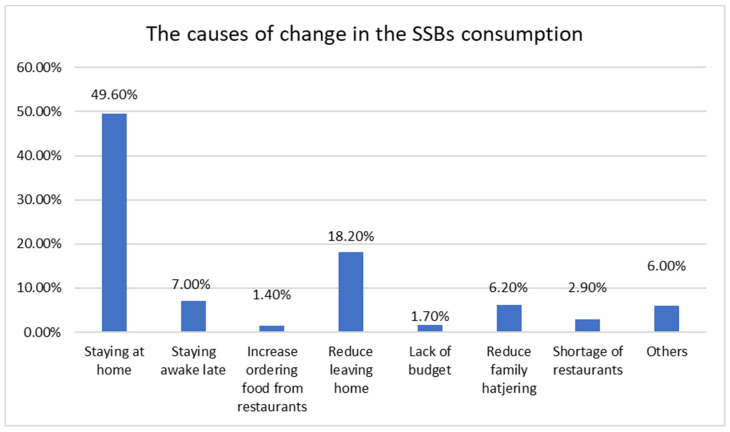
The reasons for the change in SSBs children’s consumption during the COVID-19 lockdown (N = 417).

A total of 417 parents agreed to participate and signed the consent form. Table 1 represents the socio-demographic characteristics and dental health related factors of the included participants. Table 2 describes the reported changes in the child’s SSBs consumption during the COVID-19 lockdown. Three-hundred and six participants (73.4%) reported no change in their child’s consumption of soft drinks; 53 participants (12.7%) reported an increase in soft drink consumption; and 58 (13.9%) reported a decrease in their child’s soft drink consumption. Half of responded parents reported that staying at home 209 (50.11%) was the most common reason for this change in their children SSBs consumption (Figure 2). Table 3 shows the results of the bivariate analysis between the sociodemographic and dental health related factors and the change in SSBs consumption in children during the COVID-19 lockdown in Saudi Arabia. A history of dental pain during the last year was significantly related to SSBs consumption (Pearson 0.14, *p* value = 0.004).

Additionally, we found a weak positive correlation (Pearson 0.14, *p* value = 0.003) between the consumption of SSBs and the cause of this consumption, as 42 parents reported staying at home as a factor to increase their children’s consumption of SSBs, while only 28 parents reported staying at home as a factor to decrease the consumption of SSBs. 

Table 4 shows the results of the two binary regression models. The results showed an inverse association between the history of dental pain within the last year and the SSB consumption during the COVID-19 lockdown (AOR:0.64; *p* = 0.001). The results of the multivariate regression are presented in (Appendix C), the findings were in agreement with the results of the binary logistic regression that with every unit increase in history of dental pain within the last year there was positive odds of decreased SSBs consumption among children (AOR:1.50; *p* = 0.002) during the COVID-19 lockdown.

### 3.2. Results of the Second Phase (Interview)

Themes of participants that reported an increase in their child’s SSBs consumption.

**Figure 3:**
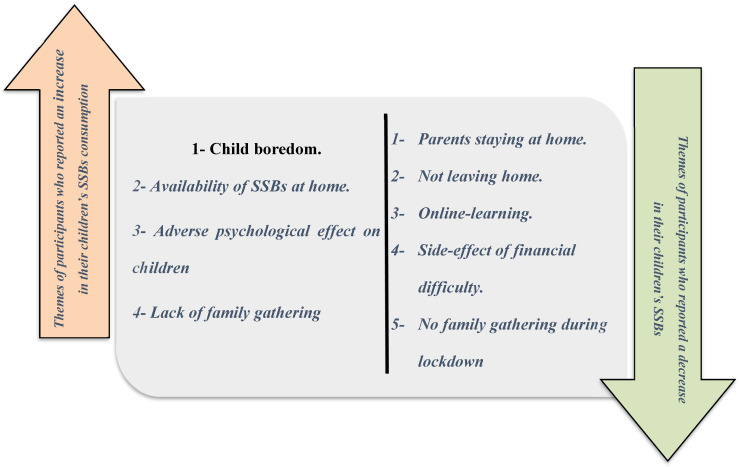
Distinctive parental themes regarding their child’s sugar-sweetened beverage consumption.

Distinctive parental themes relating to their child’s SSB consumption are shown in Figure 3.

1. Child boredom

Participants that reported an increase in their child’s consumption said that during lockdown children felt forced to stay at home, which made them bored. Some quotes from mothers illustrate this point: 

“With sitting at home and the difference in work, the boredom increased; when they are bored, the father buys them sweet drinks or they go and buy them during their free time “ (mother of child 2).

2. Availability of SSBs at home

Participants that reported an increase in their child’s consumption of SSBs said that during lockdown, SSBs were more readily available since children were usually at home. Some parents noted that their child did not ask permission to have the drink owing to its availability, which allowed them to drink more frequently. The following responses from mothers were common in this respect:

“I provide [the sweet drinks] to them, and my husband’s mother also provides the drinks, so there is no control over [use]“ (mother of child 5).

3. Adverse psychological effect on children

A: No physical activity.

Some participants mentioned that before lockdown their child used to engage in routine physical activity outside the home, such as playing football or going to the gym. They pointed out that this decrease in physical activity might have made their child become more stressed, which can lead to an increase in SSBs consumption. The following quotes from mothers demonstrate this point:

“He is a participant in the local football academy. With the start of the coronavirus, the academy closed. This was one of the reasons he was drinking sweet drinks while sitting at home” (mother of child 1). 

B: Mothers’ compensation for lockdown adversity by providing more treats.

During the lockdown children were often not able to meet friends or relatives and stayed at home. Some parents said that they felt the need to compensate for the psychological effect this had on their children by providing them with treats, including SSBs. A number of quotes from mothers support this finding:

“With boredom, their father would provide [SSBs] for them every time he went out, and the father drank [SSBs] with [the children]” (mother of child 2).

4. Lack of family gathering

One of the regulations of lockdown in Saudi Arabia was the prohibition of social gathering and the implementation of social distancing. This may have had an adverse effect on children’s psychology, which in turn may have increased consumption of SSBs. Indeed, some participants reported that the decrease in family gathering routines during lockdown affected children negatively and may have increased SSBs consumption. Example of quotes from mothers to support this theme: 

“The lack of family gatherings affected his psychology, because he did not see his relatives and became bored; these sugary drinks make up his day” (mother of child 4). 

Themes of participants that noticed a decrease in their child’s consumption.

5. Parents staying at home.

Some working mothers with long working hours reported that prior to lockdown they often did not have time to cook at home or control their child’s diet. However, as the working hours during lockdown became shorter or was carried out online, they were able to ensure suitable dietary behavior. Some reports of mothers support this point:

“Consumption decreases during [lockdown] because I’m doing other [things].” “I have more time to make fresh drinks.” “Prepare fresh drinks before they order frozen juices from outside” (mother of child 6).

6. Not leaving home.

Some parents reported that during lockdown no one was allowed to leave home without their permission. This may have constrained children in the purchasing of SSBs. Indeed, children were not able to consume SSBs whenever they had the desire for them. Some reports illustrate this theme: 

“The main reason for reduced consumption is the inability to go out of the house.’’ ”We were not going out so there is no possibility to buy it” (mother of child 10).

7. Online-learning.

Some mothers mentioned that during lockdown schooling become online and involved a marked change in their child’s daily routine. They said that normally children would go to school and could buy SSBs from the canteen. Thus, they can choose what they eat or drink, which may result in an increase in consumption of SSBs. Some quotes from mothers elucidate this theme:

“He drinks more sweetened drinks at school because they are available for him daily. The current situation at home is there are other alternatives.” “ The biggest reason for less consumption [of SSBs] is that there is no school” (mother of child 7).

8. Side-effect of financial difficulty.

Some participants pointed out that financial reasons were one of the main challenges during the COVID-19 lockdown, which influenced shopping habits. Some parents reported that their companies reduced salaries by up to a quarter of pre-COVID-19 levels, which affected total family income considerably. A number of reports from mothers relate to this point:

“Certainly, consumption decreased due to the situation during the coronavirus, owing to expenses and other conditions” (mother of child 8). 

”Her father was sitting at home; they made him redundant.” “There was no income except from me” (mother of child 8).

9. No family gathering during lockdown.

Some mothers suggested that the absence of family gathering during lockdown decreased their child’s consumption of SSBs. This was found in a number of reports:

“The lack of family gatherings, the lack of sweets and sugars.” “They drink sweetened drinks on weekends during family gatherings and go out” (mother of child 7).

## 4. Discussion

This mixed-methods study assessed the impact of the COVID-19 lockdown on SSBs consumption among 6–11 years old healthy children in Saudi Arabia and explored factors that may have influenced changes in their consumption. The study included a total of 417 children. The analysis showed considerable polarization in the trends of SSBs consumption during lockdown. The findings partly cohere with those reported by one previous study, conducted in Poland by Sidor and Rzymski in 2020, and evaluated dietary choices and habits during the COVID-19 lockdown. A significant percentage of individuals experienced modification in their dietary habits, eating and snacking more often was reported [19]. However, many participants of the present study suggested also that the lockdown actually lowered their child’s consumption of SSBs, which is a surprising result. 

Additionally, a mixed methods study [25] in the UK assessed the change in food choice motivations over lockdown among families. This study showed that lockdown for some families represented unhealthy food choices and an increase in ‘junk’ and takeaway food. These findings agreed with the resulting themes among parents who reported an increase the SSB consumption during the lockdown. 

Moreover, a newly published scoping review [26] discussed the changes in dietary habits during the COVID-19 lockdown. They reported that the COVID-19 lockdown had an impact on dietary habit practices both in a negative and positive way. Some studies found that the lockdown was associated with an increase in the number and frequency of snaking and an increase in comfort foods. while other studies found that the COVID-19 lockdown was associated with more home cooking and a reduction in comfort food and alcohol consumption. 

Globally, during COVID-19 lockdown several measures were implied to control the epidemic including travel restrictions and border closure and this had a great impact on the transport and distribution of the food supply. The Food and Agriculture Organization (FAO) stated that the COVID-19 pandemic has caused disruptions in food chains, which affecting both supply and demand [27]. Additionally, commercial determinants of health have been considered essential in the evaluation of the increase in the SSBs consumption [28]. Advertising and marketing especially among children level, have been considered important in stimulating the consumption of sugary drinks [29]. However, in the reported themes, these factors were not described by any of the participants.

The findings show a complex interaction between the child’s habits and parental, social, and financial factors during COVID-19 lockdown that affected eating and drinking habits. The main themes reported by the parents of children who saw an increase in their child’s SSBs consumption were child boredom and parental compensation for the adversity of COVID-19 lockdown. These themes were consistent with the findings of Philippe et al. (2021), which showed that child boredom and parental motivation for buying foods increased their consumption [30]. Some other themes that emerged in this study were the availability of SSBs at home, no physical activity, and the lack of family gathering. This agrees with the study conducted in 2021, by Alshehri and Al Agha, that investigated the impact of the COVID-19 lockdown on the dietary habits and physical activity of children and adolescents living in Saudi Arabia and reported an increase in the consumption of unhealthy food and drink and a decrease in the physical activities [31]. 

The main themes reported by parents of children who decreased their SSBs consumption during the COVID-19 lockdown were staying at home, online learning, and the absence of family gathering. These circumstances reportedly gave parents more time to control their child’s eating habits. These findings agree with Pujia et al. (2021), which reported a decrease in SSBs consumption among children during lockdown owing to lack of access [32]. Other themes reported by parents were the side-effect of financial difficulty and the restriction of family gathering. Alhareky et al. (2021) reported that a side-effect of financial difficulty (but not during lockdown) was reduction in SSBs consumption, which affected nearly 8% of Saudi Arabian schoolchildren [33]. 

There is a broad range of evidence across disciplines that suggests that the determinants of sugar intake in children are socioeconomic, parental, and child-related [34]. This partly supports the themes that emerged in the present study, since the COVID-19 lockdown influenced parents in various ways. Although some parents reported a decrease in SSBs consumption and others reported an increase. The socio-demographic data shows that 344 mothers (82.4%) had either a secondary or a university degree, which might have had an impact on children’s SSBs consumption because they could have informed children of the adverse consequences of these products. However, the regression analysis does not show any significant relationship with the parents’ level of education. The results of the regression analysis showed an inverse association between the history of dental pain and the consumption of SSBs. Previous studies showed that teeth pain experience is positively associated with daily soft drink consumption [35]. However, during the COVID-19 lockdown, the accessibility to dental clinics was compromised which might explain this decrease in consumption. 

In Saudi Arabia, one previous study showed that approximately 56.3% of 7–12 years old children consume carbonated beverages weekly. One of the main reasons children consume SSBs is their accessibility in school through vending machines and canteens [36]. As noted above, during the Saudi Arabian lockdown, children engaged in online schooling, which aided reduction in consumption.

In agreement with the present findings, Łuszczki et al. (2021), in a study based in Poland, showed that the frequency of SSB consumption decreased during lockdown owing to social restrictions and remote learning [37]. In our findings, there was a high percentage of parents who reported no change in their children’s consumption of SSBs and it is possible that parents are not aware of SSB consumption at school, and some children in the no change group may have a decreased consumption. This finding suggests the need for school-based interventions that can enhance awareness of the impact of providing SSBs in schools and provide healthy alternatives, such as milk and water.

One of the strengths of this study is its design, since both a quantitative questionnaire and qualitative interviews permit a holistic overview of the impact of the COVID-19 lockdown. The interviews in the questionnaire component involved open-ended questions and conversational communication with the child’s parent, providing descriptive, valid, and understandable results [38]. A triangulation of different methods increased the research validity and depth of information. Additionally, several methods were used to establish the creditability of the study, such as multiple coders and engagement with the participants [39]. Further, the study complied with the consolidated criteria for reporting qualitative research (COREQ) checklist [40].

The limitations of the study were the potential for the introduction of subconscious bias and conclusions of the interviewer, which may have influenced the emergence and interpretation of the themes. There were no in-depth interviews conducted with those who had no change in SSBs consumption. In general, there is a potential for bias in the researcher and presumptions when carrying out a study. As part of the bracketing process, one researcher (AH) that was experienced in practicing qualitative methodology discussed the research process and findings with a qualitative researcher (KB) to overcome any bias and presumptions that may have been present. Other challenges include losing track during interviews, steering the direction of conversation, inadvertently inhibiting participants, and not allowing clarity in the preparation and presentation of questions. To counter these issues, deliberate reflexive practice was employed, enabling improved interview technique. Other limitations may be related to the purposive sampling, which can result in sampling bias [41]. 

## 5. Conclusions

This study showed the impact of the Saudi Arabian COVID-19 national lockdown on children’s consumption of SSBs as reported by parents. A relatively small change in the consumption of all types of SSBs was reported. The results showed an inverse association between the history of dental pain during the last year (AOR:0.64; *p* = 0.001) and SSBs consumption during COVID-19 lockdown. Different themes that influenced differential consumption of children were described by the parents including: child boredom, parental compensation for lockdown adversity, adverse psychological effect on children, online schooling, family gathering, and the side-effects of financial adversity. The study highlights the need to challenge negative influences and school-based interventions. The study’s findings highlight the need for interventions that can enhance awareness of SSB consumption that target both parents and children to aid the development of healthy drink substitutes.

## Figures and Tables

**Table 1:** The demographic characteristics of participants.

Socio-Demographic Variable	Number (n = 417)	Percentage (100%)
Child nationality		
Saudi	373	89.4
Non-Saudi	44	10.6
Participant		
Mother	348	83.4
Father	30	7.2
Other	39	9.4
Marital status		
Married	381	91.4
Single/Divorced	36	8.6
Mother’s Level of Education		
Less than Secondary	14	3.4
Secondary/University	344	82.4
Higher education	59	20.6
Father’s Level of Education		
Less than Secondary	31	7.4
Secondary/University	300	71.9
Higher education	86	17.7
Number of Children		
One	32	7.7
Two	100	24
Three	101	24.2
Four or more	184	44.1
Monthly Family Income		
High > 1000 SR	224	53.7
Moderate 7000–10000 SR	117	28.1
Low < 7000 SR	76	18.2
Age of participant parent		
<20	19	4.6
20–30	90	21.6
31–40	195	46.7
>40	113	27.1
Child Lives with		
One parent	26	6.2
Both parents	385	92.3
Others	6	1.4

**Table 2:** Lockdown in Saudi Arabia.

SSBs Type	Increase		Decrease		Child does not Consume SSBs/no Change		Total N = 417
	Frequency	%	Frequency	%	Frequency	%	Total%
Soft Drinks	53	12.7	58	13.9	306	73.4	100
Fruit Juice	87	20.8	115	27.6	215	51.6	100
Flavored Milk	63	15.1	93	22.3	261	62.6	100

SSBs: Sugar-sweetened beverages.

**Table 3:** Bivariate analysis showing the correlation between the sociodemographic and dental factors and the change in soft drinks consumption in children during the lockdown in Saudi Arabia.

Socio-Demographic Variable N = 417	Increase		Decrease		Child does not Consume SSBs/no Change		*p*-Value *
	Frequency	%	Frequency	%	Frequency	%	
Mother’s Education Level							0.56 ^$^
Less than Secondary	7	1.67	1	0.23	6	1.43	
Secondary/University	179	42.92	52	12.47	113	27.09	
Higher education	34	8.15	5	1.19	20	4.79	
Father’s education level							
Less than Secondary	11	2.63	6	1.43	14	3.35	0.10
Secondary/University	153	36.69	45	10.79	102	24.46	
Higher education	56	13.42	7	1.67	23	5.51	
Parents marital status							
Married	200	47.96	52	12.47	128	30.69	0.66
Single/Divorced	20	4.79	6	1.43	11	2.63	
Family average monthly income							
High >1000 SR	44	10.55	10	2.39	22	5.27	0.96
Moderate 7000–10000 SR	59	14.14	18	4.31	40	9.59	
Low < 7000 SR	117	28.05	30	7.19	77	18.46	
Child lives with whom							
One parent	5	1.19	1	0.23	2	0.47	0.76 ^$^
Both parents	206	49.40	54	12.94	128	30.69	
Others	9	2.15	3	0.71	9	2.15	
Visiting the dentist last year							
Yes	144	34.53	41	9.83	90	21.58	0.78
No	76	18.22	17	4.07	49	11.75	

* Chi-square test; ^$^ Fishers exact test (cells has less than 5).

**Table 4:** Binary logistic regression model to determine factors associated with children’s SSBs consumption during the lockdown.

Variable		Increased SSBs (n = 53) ^$^		Decreased SSBs (n = 58) ^$^	
		AOR	*p* Value	AOR	*p* Value
Mother’s level of education	Less than secondary	0.49	0.55	1.24	0.88
	Secondary/University	0.54	0.36	0.69	0.58
	Higher education	1.00		1.00	
Father’s level of education	Less than secondary	0.62	0.58	0.51	0.46
	Secondary/University	0.81	0.73	0.65	0.48
	Higher education			1.00	
Parents marital status	Married	2.48	0.21	2.1	0.31
	Single/Divorced	1.00		1.00	
Family average monthly income	Low income	1.11	0.85	1.04	0.93
	Moderate	1.14	0.78	1.08	0.85
	High	1.00		1.00	
Child lives with whom	One parent	0.62	0.99	0.74	0.85
	Both parents	0.47	0.39	0.50	0.38
	Others	1.00		1.00	
Visiting the dentist last year	Yes	0.72	0.44	0.78	0.54
	No	1.00		1.00	
History of pain in the last year		0.81	0.14	0.64	0.001 *

^$^ Does not change is the reference group (n = 87); * *p* value significant < 0.05.

## Data Availability

Not applicable.

## Appendix A

**Questionnaire included questions regarding any changes in the child’s consumption of SSBs during the lockdown**

Dear parent,

We are a researcher’s team, conducting a study on the effect of COVID-19 pandemic lockdown on children’s consumption of sugary drinks.

If you have a healthy child between 6 and 11 years old, please fill out the questionnaire (If you have more than one child within this age group, please fill out the questionnaire based on the youngest child).

The survey results will help us assess the health benefits and harms of children consuming sugary drinks after this pandemic.

There are no right or wrong answers in this questionnaire and all information provided will be treated as strictly confidential.

Questions of the demographic characteristics of the participants:

1- What is the nationality of the child?

Non-Saudi   Saudi

2- What is the relationship with the child?

Other   Father   Mother

3- How old are you?

More than 40   31–40   20–30   Less than 20

4- What is the marital status?

Other   Separated/Divorced   Single   Married

5- What is the mother’s level of education?

Higher education   University   Secondary   Primary   Not educated

6- What is the father’s level of education?

Higher education   University   Secondary   Primary   Not educated

7- Number of Children?

More than three   Three   Two   One

8- What is the monthly family income?

Low (less than 7000)   Moderate (7000–10,000)   High (more than 10,000)

9- What is the age of your child

10- How much does a child weight in Kg?

11- How tall is the child in Cm?

12- Who does the child live with?

Other   Father and stepmother   Mother and stepfather   Mother and Father   Father only   Mother only

Questions about child’s visits to the dentist and dental pain suffering, with general questions about the child’s teeth and health:

13- Have you ever taken your child to the Dentist?

- Yes
- No
- I do not know
- I did not remember

14- Has the child complained of any dental pain during the past year?

- The child did not complain of toothache
- Yes, once
- Yes, Twice
- Yes, three times
- Yes, more than three times
- I do not know
- I did not remember

15- When one of your children complains of toothache, do you:

- Give the child a sedative
- Give the child some antibiotics
- Go to the Dentist
- Go to the Pharmacist
- Seek other medical care
- Use herbal medicine
- You ask the child about the tooth that hurts until you take it out
- Family consulting
- Do nothing, and often get better on their own
- None of my children have ever complained of toothache before

Questions about the changes in SSBs consumption among the children during lockdown in Saudi Arabia:

16- Did your child’s consumption of soft drinks changed during the lockdown?

Yes, Increase   Yes, Decrease   No change   Child not consumed SSBs

17- Did your child’s consumption of Juices changed during the lockdown?

Yes, Increase   Yes, Decrease   No change   Child not consumed SSBs

18- Did your child’s consumption of Milk with flavors changed during the lockdown?

Yes, Increase   Yes, Decrease   No change   Child not consumed SSBs

19- If there is a change in the consumption of the child during the lockdown (whether its increases or decreases), please choose the reason (more than on potion can be selected):

- Stay at home
- Stay up late
- Increase ordering food from restaurants
- Reduce leaving home
- Lack of budget
- Reduce family gathering
- Shortage or restaurants
- Other

20- If the answer in the previous question was due to other reason, please clarify them?

21- Does taxation affect your child’s consumption of SSBs?

- Yes
- No

-In case we need to contact you for the second phase of the study (interviews), can you write the contact number please (this is completely optional)

## Appendix B

**Topic guide used during the structured interviews**

Hello, my name is   I’m a dentist

This interview scheduled to find out what do you think about your child sugar consumption during the COVID-19 lockdown.

Assure confidentiality

“We want to hear what you do and what you think. Every family is different and there are no right or wrong answers. We are not here to judge you as a parent, so please feel to talk openly and honestly. We will keep what you say strictly confidential. “

Explain the format of the interview and audio recording

Indicate how long the interview will take (approximately 30 min)

There are no right or wrong answers. We would really like to hear what everyone has to say.

**Section 1** **: Information about parents**

- Tell me about yourself?
- How old are you?
- Are you married?
- Tell me about your education? and what is your academic major? and what is the last degree certificate you obtained?
- Are you working? What is your occupation (job)? How many hours do you spend on your work?
- How many kids do you have?
- Who is taken care of your children during your working hours? Is this changed during a lockdown?
- Tell me about the place you are currently live in?
- Are you live with your family in a big house or a small one?
- Is it the same building that your family lived in?
- Do you have a regular family gathering that has a certain place and time?
- Tell me about your work during the lockdown, was it online or not?

**Section 2** **: Information about child**

- What is your child’s age?
- Tell me about your child’s oral health?
- What about tooth brushing? Prompts (regular, before bedtime)
- How many times did your brush his\her teeth?
- What about dental visits? Prompts (regular, fluoride)
- What are the reasons for a child’s dental visit usually?
- Tell me about your child dietary habit? Who made the decision of what to buy or eat and cook?
- What do you think about your child diet? healthy diet or not? and tell me about the snacks and meals?
- What is the content of meals and snacks?
- Let us now speak about sugar sweetened beverages (SSBs)? And when your child consuming these drinks? prompts (weekday or weakened, with meals or between meals, at home or with family gathering).
- What about food shopping? and who is responsible for the grocery for home, is it you or the father? Do you take him with you? You let him pick what he wants?
- Tell me about buying SSBs? Is it the child picking from the grocery shelf or it is your choice? Do you buy one piece or cartoon?
- What is the favorite choices drinks of your child during shopping? and who is made the decision, is it yours or your child?
- Tell me more about availability of SSBs consumption on the home?
- Are SSBs usually available at home for the child? Is it a child’s choice?
- Does anyone in your family like to drink SSBs (his brother, his father)? Is he sharing the drink with others or father?
- What is the frequency of consuming SSBs? Is it daily, or at weekend? is it only on occasion and gathering?

**Section 3: Changes during the lockdown**

Finally, tell me more about the lockdown period?

It was very hard time? What do you think about grocery shopping? was it affected?

- During the lockdown period: (total and partial lockdown hours)
- Was SSBs always available at home?
- What do you think more consumption of SSBS or less during the lockdown? And why?
- What about sharing the drink with others or father during the lockdown?
- How much time he is consumed the SSBs? Is it daily, or at weekend? is it only on occasion and gathering?
- Why do think about the change in the consumption during lockdown? Prompts (decrease, increases, family gathering- normal shopping- financial reasons- treats during lockdown)

**Last part about the lockdown period and restriction policies:**

- Has the consumption of SSBs changed? Why does SSB’s consumption of your child increase or decrease during a lockdown?
- What is the main reason for this change?
- What do you think about absence of parties and family gatherings during lockdown?
- What about family gatherings? Tell me more about this.
- What do you think about restriction of the accessibility to markets and shopping centers?
- What do you think about financial problems during the lockdown? Prompts (some families were affected financially during the lockdown)

## Appendix C

**Table A1:** Multinominal logistic regression model to determine factors associated with children’s SSBs consumption.

Variable		Increased SSB			Decreased SSB			Child Not Consuming		
		AOR	95% CI	*p* Value	AOR	95% CI	*p* Value	AOR	95% CI	*p* Value
History of pain in last year		1.13	0.88–1.46	0.32	1.50	1.18–1.92	0.001 *	0.82	0.67–1.01	0.07
Mother education	Less than secondary	1.99	0.21–18.5	0.54	0.43	0.27–7.03	0.56	1.73	0.30–9.82	0.53
	Higher than secondary	1.81	0.48–6.85	0.37	1.66	0.42–6.41	0.46	1.82	0.77–4.28	0.16
	Higher education	1.00			1.00			1.00		
Father education	Less than secondary	1.38	0.27–6.96	0.69	1.28	1.27–7.09	0.69	0.31	0.08–1.08	0.06
	Higher than secondary	1.15	0.34–3.84	0.81	1.07	0.31–3.66	0.90	0.56	0.25–1.24	0.15
	Higher education	1.00			1.00			1.00		
Child visit to the dentist	Yes	1.27	0.58–2.77	0.53	1.11	0.51–2.4	0.77	1.21	0.69–2.13	0.49
	No	1.00			1.00			1.00		
Marital status	Married	0.38	0.10–1.45	0.15	0.39	0.10–1.50	0.17	0.62	0.21–1.84	0.39
	Single/Divorced	1.00			1.00		1.00			
The child lives	One parent	6.42	0.00–6.89	0.99	0.95	0.53–17.12	0.97	1.67	0.22–12.37	0.61
	Both parents	2.46	0.47–12.83	0.28	1.6	0.37–7.15	0.50	2.17	0.75–6.28	0.15
	Others	1.00			1.00			1.00		
Monthly income	Low income	0.79	0.27–2.11	0.67	0.73	0.25–2.08	0.55	1.73	0.79–3.79	0.17
	Moderate	0.85	0.37–1.95	0.70	0.86	0.38–1.97	0.73	1.05	0.57–1.93	0.87
	High	1.00			1.00			1.00		

* Significant level less than <0.05.
